# *In vitro* biological assessment of *berberis vulgaris* and its active constituent, berberine: antioxidants, anti-acetylcholinesterase, anti-diabetic and anticancer effects

**DOI:** 10.1186/1472-6882-13-218

**Published:** 2013-09-05

**Authors:** Abeer E Abd El-Wahab, Doaa A Ghareeb, Eman EM Sarhan, Marwa M Abu-Serie, Maha A El Demellawy

**Affiliations:** 1 Biochemistry Department, Faculty of Science, Alexandria University, P.O. Box: 21511, Alexandria, Egypt; 2 Medical Biotechnology Department, Genetic Engineering & Biotechnology Research Institute, City for Scientific Research & Technology Applications, Alexandria, Egypt; 3 Protein research Department, Genetic Engineering & Biotechnology Research Institute, City for Scientific Research & Technology Applications, Alexandria, Egypt

**Keywords:** Bioscreening, DPPH, Acetylcholinesterase, α-glucosidase, Breast cancer, Hepatoma, CACO-2, PBMC

## Abstract

**Background:**

*Berberis vulgaris* is a well known plant with traditional herbal medical history. The aims of this study was to bioscreen and compare the *in vitro* biological activity (antioxidant, cholinergic, antidaibetic and the anticancer) of barberry crude extract and berberine active compound.

**Methods:**

The effect of *B. vulgaris* extract and berberine chloride on cellular thiobarbituric acid reactive species (TBARS) formation, diphenyle–α-picrylhydrazyl (DPPH) oxidation, cellular nitric oxide (NO) radical scavenging capability, superoxide dismutase (SOD), glutathione peroxidase (GPx), acetylcholinesterase (AChE) and α-gulcosidase activities were spectrophotometrically determined. On the other hand, the effect of extract and berberine as anticancer was estimated on three different cell lines which were MCF-7, HepG-2, and Caco-2 cells by using neutral red uptake assay which compared with control normal cells (PBMC).

**Results:**

Our results showed that barberry crude extract contains 0.6 mg berberine/mg crude extract. Barberry extract showed potent antioxidative capacity through decreasing TBARS, NO and the oxidation of DPPH that associated with GPx and SOD hyperactivation. Inhibitory effect of *berberis* crude extract on α-glucosidase was more potent than that of berberine chloride, while both had the same AChE inhibitory effect. Besides, different concentrations of both berberine chloride and barberry ethanolic extract showed to have no growth inhibitory effect on normal blood cells (PBMC). Otherwise, both berberine chloride and barberry ethanolic extract showed to have inhibitory effect on the growth of breast, liver and colon cancer cell lines (MCF7, HepG2 and CACO-2, respectively) at different incubation times starting from 24 hrs up to 72 hrs and the inhibitory effect increased with time in a dose dependant manner.

**Conclusion:**

This work demonstrates the potential of the barberry crude extract and its active alkaloid, berberine, on suppressing lipid peroxidation, suggesting a promising use in the treatment of hepatic oxidative stress, Alzheimer and idiopathic male factor infertility. Beside*, berberis vulgaris* ethanolic extract is safe non-toxic extract as it was not inhibit the growth of PBMC that can induce cancer cell death that could return to its powerful antioxidant activity.

## Background

As it is increasingly believed now that traditional medicines become more popular worldwide, there is accumulating evidence suggesting medicinal plants are unlimited reservoirs of drugs. The amazing structural diversity among their active components makes them a useful source of novel therapeutics. Researchers with interest in natural products have intensified their efforts towards scientific evaluation of traditional medicines. The World Health Organization (WHO) estimates that herbal medicine is still the most common source for primary health care of about 75-80% of the world's population, mainly in the developing countries, because of better cultural acceptability, better compatibility with the human body and fewer side effects [1].

*Berberis vulgaris* is a shrub in the family Berberidaceae, native to central and southern Europe, northwest Africa and western Asia. It grows in a variety of soils, though is primarily cultivated in cooler regions [2-4]. Its fruit is an oblong red berry 7–10 mm long and 3–5 mm broad, ripening in late summer or autumn; it is edible but very sour, and rich in vitamin C. Barberry is extensively used as food additive and its juice is recommended to cure cholecytitis [5].

Barberry has a long history of use in traditional eastern and western herbalism [6].

*Berberis vulgaris* as well as other berberine (BER) containing plants [7] are used medicinally in virtually all-traditional medical systems, and have a history of usage in Ayurvedic, Iranian and Chinese medicine dating back at least 3,000 years [8]. Ancient Egyptians used barberry fruit with fennel seeds to ward off pestilent fevers [6]. Indian ayurvedic physicians used barberry in the treatment of dysentery and traditional Iranian medicine uses its fruit as a sedative [6,9]. In northern Europe barberry was used to treat gall bladder and liver problems, while it was used in the treatment of abnormal uterine bleeds and rheumatism in Russia and Bulgaria [10,11]. In North America, the Eclectics used barberry for treatment of malaria and as a general tonic [12]. Also, the American Indians found it effective in improving appetite and used its dried fruit as a gargle [13,14].

Phytochemical analysis of root, stem or bark extract of *B. vulgaris* demonstrated the presence of protoberberines and bisbenzyl-isoquinoline alkaloids (berbamine, tetrandrine and chondocurine, (Table 1) for which anti-inflammatory and immuno-suppressive activities have also been well established [15]. Medicinal properties for all parts of the plant have been reported, including tonic, antimicrobial, antiemetic, antipyretic, antipruritic, antioxidant, anti-inflammatory, hypotensive, antiarrhythmic, sedative, antinociceptive, anticholinergic and cholagogue actions, and it has been used in some cases like cholecystitis, cholelithiasis, jaundice, dysentery, leishmaniasis, malaria and gall stones [16] (Table 2). Furthermore, BER, an isoquinoline alkaloid and the major ingredient of this plant, has been used for treating diarrhea and gasterointestinal disorders for a long time [17,18]. It has multiple pharmacological effects including; antimicrobial activity against 54 microorganisms [19-21], inhibition of intestinal ion secretion and smooth muscle contraction, inhibition of ventricular tachyarrhythmia, reduction of inflammation, stimulation of bile secretion and bilirubin discharge [22]. In spite of extensive applications and numerous properties, the mechanism of action in most of its effects is not exactly clear. Some of these properties may occur due to antihistaminic and anticholinergic effects.

**Table 1:** Compounds isolated from berberis vulgaris

**Compound**	**Nature**	**Compound**	**Nature**
Aromoline	Alkaloid	Oxyberberine	Alkaloid
Berbamine	Alkaloid	Oxycanthine	Alkaloid
Berberine	Alkaloid	Palmatine	Alkaloid
Berlambine	Alkaloid	Quercentin	Flavonoid
Bervulcinc	Alkaloid	Rutin	Flavonoid
Columbamine	Alkaloid	(-)-tejedine	Alkaloid
Hydroxycanthine	Alkaloid	Yatronizine	Alkaloid
Isocorydine	Alkaloid		

**Table 2:** **The pharmacological effects of** ***Berberis vulgaris*** **(NAPALERT; natural products alert database)**

**System**	**Effect**	**Part of plant**	**Preparation**
Cardiovascular	Hypotensive activity	Dried root	Alkaloid fraction
		Dried fruit	Aqueous extract
Gastrointestinal	Gastric secretory stimulation	Root	Ethanol—H_2_0 (67%) extract
Endocrine	Choleretic activity in rat	Dried root	Total alkaloids
	Choleretic activity	Stem bark	
	Increases tone of the digestive tract and gives rise to increased and irregular peristalsis	Dried root	
	Anticholinergic activity in guinea pig ileum	Dried fruit	Decoction
	Menstruation induction effect in guinea pig	Stem	Ethanol (95%) extract
	Uterine stimulant effect in cat, rabbit and guinea-pig	Leaf	Ethanol-acetone (50%) extract
Immune system	Antibody formation suppression in mouse	Dried root	Alkaloid fraction
	Antiinflammatory activity	Root	Alkaloid fraction
Organisms	Complement alternative pathway inhibition	Root	Ethanol (l00%) extract
	Delayed type cutaneous hypersensitivity inhibition		Alkaloid fraction and ethanol (95%) extract Alkaloid fraction
Central nervous system	Antipyretic activity in rat	Dried bark	Alkaloid fraction
		Dried fruit	Ethanol (95%) extract
	Narcotic antagonist activity	Dried root	
	Sedative	Fruit	
Renal	Diuretic activity in rat	Dried bark	Alkaloid fraction
Other	Toxicity assessment in mouse — LD_50_ = 520 0 mg/kg	Dried root	Alkaloid fraction
	Toxicity assessment in mouse — LD_50_ = 2.6 ± 022 g/kg b w		
Male reproduction	Idiopathic ma factors due to oxidative damage	Root	Crude methanolic (95%) extraction
			Crude acetic acid (5%) extraction

Among berberine multiple pharmacological actions, anti-inflammatory activity has been extensively studied [23]. Antipyretic activity of berberine sulfate has also been shown by Sabir and Bhide 1971 using a model of experimentally induced fever in rats [22]. This effect has been found to be approximately three times greater than sodium salicylate. Anti-colitic property is another pharmacological effect has been demonstrated for berberine by Zhou and Mineshita [24].

The barberry phenolic compounds include anthocyanins and carotenoid pigments [22-26]. Several pharmacological effects such as antioxidant and cytoprotective [27], inhibitory effects on capillary permeability [28] and epidermal growth factor [29] anticholinergic and antihistaminergic [27], have been demonstrated for anthocyanins and berberry fruit extract (BFE).

The aim of this study was to compare the *in vitro* biological activity (antioxidant, cholinergic, antidaibetic and the anticancer) of barberry crude extract and berberine active compound.

## Methods

### Human and animal biological samples

Human participants and their biological materials (blood) met the ethical standards for donor approval required by national regulatory bodies. Blood samples were collected from ten healthy subjects after they signed consent informed the use of their blood in this study Consents’ approval and all study protocols for animal and biological tissue samples treatment, involved in this study, were firmly subjected to ethical instructions outlined by Animal Ethics Committees (AEC) that published via The National Health and Medical Research Council (NHMRC) policies and guidelines that recommended by the Egyptian Ministry of Health and Population, High Committee Of Medical Specialties, Arab Republic of Egypt [30]. This study was permission granted from the Biomedical technology, SRAT-city and Biochemistry Department, Faculty of Science, Alexandria University, following approval of the Research Ethics Committee, Faculty of Pharmacy, Alexandria University.

### Barberry, *Berberis vulgaris*

Barberry’s roots were purchased and authenticated by Prof. Salma El-dareir, Botany Department, Faculty of Science, Alexandria University, Egypt. Firstly, this classification was being dependent on the data about the plant published in Dargon Herbarium [31]. After classification, the plant roots were separated then dried at room temperature, powdered, sieved, and stored prior to further use. Dried barberry roots were phytochemically screened for alkaloids, phobatannins, saponnins, flavonoids, steroids, terpenoids and cardiac glycosides [1].

### Barberry crude extract preparation

The dried powdery roots of barberry were exhaustively defatted with petroleum ether then dried in fresh air to evaporate the solvent. The dried roots were used to prepare the ethanolic crude extract by subjecting to steam distillation method using Soxhlet apparatus in which the powder was added in glass thimble and boiled ethanol extracted the active compounds for 8 hours. The ethanolic extract was concentrated to minimum volume using rotary evaporator at 60°C and 100 rpm (Büchi, Switzerland) then lyophilized (DISHI, DS-FD-SH10, Xi’an Heb Biotechnology Co, China) to obtain a powder extract of barberry (25%). The barberry extract powder form was kept at -20°C until subjected to further biochemical analysis.

### HPLC analysis

The ethanolic extract was analyzed using HPLC (Series 500 Bio-Tek Instrument, Milano, Italy) to determine the berberine concentration in the extract. The analytes were separated by a Zorbax Eclipse XDB-C18 (250 × 4.6 mm i.d., 5 μm particle size) column (Agilent, Santa Clara, CA, USA).

The freeze-dried *Berberis vulgaris* extracts were dissolved in equal volume water: ethanol solution (1 mg/ml) and were filtered through a 0.22-μm syringe filter prior to HPLC analysis. The operating temperature was maintained at 30°C and the detector was operated at a wavelength of 254 nm. The mobile phase was a mixture of two solvent compositions, solvent A (deionized water) and solvent B (methanol). The program was started with 40% solvent A and 60% solvent B at a solvent flow of 0.8 ml/min and injection volume of 20 μl [32].

### Preparation of berberine chloride

Berberine chloride was commercially available from Sigma-Aldrich where, different concentrations of berberine chloride were prepared by dissolved certain weights at 1 ml of 10% ethanol.

### Preparation of liver homogenate

Six Balb/c mice were obtained from animal of house of medical research institute, Alexandria University. After anesthesia, liver was isolated and washed in cold saline, and then one gram of each liver was homogenized in 9 mL phosphate buffer saline. The homogenate was centrifuged at 3000 and metabolites containing supernatant was decanted for further biochemical estimations.

### Biochemical assays

1. Determination of Acetylcholinesterase (AChE) activity

AChE activity was measured according to the method of Ellman *et al*. [33]. 130 μL phosphate buffer (0.1 M pH 7.4) were added to a mixture of 20 μL of liver homogenate and 20 μL of barberry extract (test) or organic solvent (control), then incubated for 45 min at 37°C. 5 μL of substrate ACTI (75 mM) were added, mixed well and incubated for 15 min at 37°C. 60 μL DTNB (0.32 mM) were added and left for 5 min. The absorbance was measured at 405 nm and the specific activity was calculated.

2. Determination of α-glucosidase activity

Method mentioned by Han and Srinivasan [34] was carried out with a slight modification to estimate the effect of barberry extract on α-glucosidase (EC 3.2.1.20) activity. 100 μL of barberry extract (test), organic solvents (control) or dH2O (blank) were diluted with 2.5 mL of 0.1 M phosphate buffer, pH 7.4. 100 μL of liver homogenate were added, mixed well and incubated in a water bath with the reaction mixture at 30°C for 5 min. 500 μL PNPG, 5 mM, were added and the reaction was allowed to proceed for 15 min. The reaction was stopped by the addition of 2 mL of 1 M Na_2_CO_3_. The producing color was spectrophotometrically detected at 400 nm. A unit of enzyme activity was defined as nmoles of p-nitrophenol released/min.

3. Determination of Thiobarbaturic acid reactive substance (TBARS) level in induced lipid peroxidation model

Two mL of barberry extract or berberine chloride (test), the organic solvent (control) or distilled water (dH_2_O) (blank) were incubated with equal volume of liver homogenate for about 45 min at 37°C. *In vitro* tissue lipid peroxidation was induced by adding H_2_O_2_ and ferrous sulphate (FeSO_4_.7H_2_O) at a final concentration of 1 mM and 0.5 mM, respectively, in both test and control reaction mixtures. After an incubation period of about 30 min at 37°C, butylated hydroxyl toluene (BHT) was added at a final concentration of 0.02% and mixed carefully to stop the peroxidation reaction. The mixtures were centrifuged at 3000 rpm for 15 min, and then 1 mL of the resultant supernatant was mixed with 1 mL of 15 % trichloroacetic acid (TCA) followed by centrifugation at 3000 rpm for 10 min [1].

Then TBARS was determined in previous solution according to the method described by Wills [35]. 1 mL of protein free supernatant was mixed with 500 μL of 0.7% thiobarbituric acid (TBA), heated in boiling water bath for 45 min, cooled and the colour in the supernatant was detected at 532 nm. The TBARS level was calculated against a control according to the following equation: TBARS level (nmol/ml) = At / 0.156

4. Determination of Diphenyle –α-picrylhydrazyl (DPPH) radical scavenging

$$\mathrm{\%scavenging}=[\left(A\;\mathrm{control}-A\;\mathrm{sample}\right]/A\;\mathrm{control}\;\times \;100$$

DPPH radical scavenging assay of the total extract was performed by using the previously established and modified methodology by Choi *et al.*[36]. Assays were performed in flat bottom polystyrene 96 well microtiter plates. To 100 μL of each sample (1-6 mg/ml) in EtOH 25 μL DPPH (1 mM) in ethanol was added. The resultant mixture was briefly shaken and maintained at room temperature, in the dark for 30 min. At the end of this period, the absorbance (A) of the mixture was measured at 490 nm, using ELISA. Scavenging ratio of DPPH assay calculated as follows:

5. Determination of nitric oxide scavenging activity

$$\mathrm{NO}\;\mathrm{scavenged}=\left[\left(A\;\mathrm{control}-A\;\mathrm{test}\right)/A\;\mathrm{control}\right]\;\times 100$$

Nitric oxide was determined by the method described by Green *et al*. [37], where nitric oxide was generated from sodium nitroprusside and measured by Griess reaction. Scavenger of nitric oxide competes with oxygen leading to reduced production of nitric oxide. Sodium nitroprusside (5 mM) in phosphate buffered saline (pH 7.2), was mixed with different concentrations (1–6 mg/ mL) of the extract and incubated at 25°C. Samples from the above were reacted with Griess reagent (1% sulphanilamide, 2% phosphoric acids and 0.1% Naphthylethylendiamine hydrochloride). The absorbance of the chromophore (A) formed during the diazotization of nitrite with sulphanilamide and subsequent coupling with naphthylethylenediamine was read at 546 nm. The scavenged ratio of NO calculated as follows:

6. Determination of SOD activity

Twenty microliters of liver homogenate supernatant (test) or buffer (reference) and 20 μl of extract or berberine chloride different concentrations were added to 1 mL buffer solution and incubated at 37°C for 45 min. 10 μl pyrogallol (20 mM in HCl, 10 mM) was added to the previous solution and the absorbance of test (At) or reference (Ar) was measured at 420 nm against air after 30 and 90 s. The percentage inhibition of pyrogallol autoxidation by supernatant was calculated according to the following equation:

$$\begin{array}{c} \mathrm{The}\;\mathrm{percentage}\;\mathrm{inhibition}\\ \;=100–[\left(\mathrm{At}/\min/\mathrm{ml}\;\mathrm{sample}\right)/\\ \;\left(\mathrm{Ar}/\min/\mathrm{ml}\;\mathrm{reference}\right)\;\times \;100] \end{array}$$

The specific activity of serum SOD as ng/min/mg protein was calculated with dividing the value of SOD in ng/min/ml by protein concentration in the sample.

One unit of SOD activity is defined as the amount of enzyme which inhibits the rate of auto-oxidation of pyrogallol by 50%. From the standard curve it was found that, one unit equals 153 ng. The sample enzyme activity in U/ mg protein was obtained by diving value in ng/ min/ mg protein by 153 [38].

7. Determination of liver glutathione peroxidase (GPx) activity

Fifty microliters of liver homogenate were incubated with equal volume of different extract or berberine chloride different concentrations for 45 min at 37°C, then were added to 100 μl GSH, 100 μl cummen H_2_O_2_ and 750 μl Tris–HCl, pH 7.6 (test) and incubated at 37°C for 10 min. For control, 50 μl diluted supernatant and100 μl GSH were added to 750 μl Tris–HCl, pH 7.6, then incubated at 37°C for 10 min. One milliliter TCA was added to test and control as well as 100 μl cummen H_2_O_2_ were added to control then both were centrifuged at 3000 r.p.m. for 20 min and then the supernatants were separated off. One milliliter of supernatants was added to 2 ml Tris–HCl, pH 8.9 and 100 μl DTNB then incubated for 5 min [39]. The absorbances of test and control (Ac) were read at 412 nm against distilled water. The activity of liver GPx was calculated with the following equation;

$$\begin{array}{c} \mathrm{GPx}\;\mathrm{activity}\;\left(U/g\;\mathrm{wet}\;\mathrm{tissue}\right)\\ \;=E\times 6.2\times 10\times 10/13.1\times 0.05\times 10 \end{array}$$

### *In Vitro* anticancer assay

In order to determine the safe concentrations of both barberry ethanolic extract and berberine standard that can be used for *In Vitro* cell culture; normal peripheral blood mononuclear cells (PBMC) were isolated from a healthy individual by Ficoll-Hypaque (density 1.077 g/L, Lonza, USA) gradient centrifugation. PBMC were collected and washed using HBSS, then cell viability and count were determined using Trypan blue exclusion test. After collecting the PBMC, cells were suspended at concentration of 1×10^6^ cell/ml in RPMI 1640 (Lonza) 25 mM *N*-2-hydroxyethylpiperazine-*N`*-2-ethanesulfonic acid (HEPES) (Lonza), 4 mM L-glutamine (Lonza), 100U of penicillin and 100 μg streptomycin (Lonza) and 10% FBS (Lonza). In 96 well plate, 1×10^5^ cell/well were seeded with different concentrations ranging from 0 to 100 μg/ml (20, 40, 60, 80, 100 μg/ml) of barberry ethanolic extract, or berberine standard for 72 hr at 37°C incubator with 95% humidity and 5% CO_2_.

The cytotoxic effect of the different concentrations of the extract and the standard were determined using the neutral red uptake assay [40]. Briefly, Neutral red working solution (80 μg/ml) (Serva, Austria) was incubated overnight at 37°C. In each well of the incubated cells, culture media was removed and 100 μl of neutral red medium were added then incubated for 3 hr to allow for vital dye incorporation into living cells. The neutral red media was removed and rapid rinsed with 150 μl HBSS. Dye was extracted from the cells by adding 150 μl extraction buffer (1% acetic acid: 50% ethanol (96%): 49% deionized H_2_O) followed by rapid agitation for at least 10 min on micrometer plate shaker. The extract neutral red color intensity was measured at 490 nm in a micro-titer plate reader spectrophotometer.

Using the relation between used concentrations and neutral red intensity value, IC_50_ of the barberry ethanolic extract and standard berberine chloride was calculated.

Following the same method selected concentrations of both barberry ethanolic extract and standard berberine chloride were used to examine their effect on different cancer cell lines. In a 96-well tissue culture plates, MCF-7, HepG-2, Caco-2 and EL4 cells were plated each in its respective culture media, at a density of 3000 cells/well, 11000 cells/well, 6000 cell/well and 15000 cells/well, respectively. Cells were left to adhere by incubation for 24 hr at 37°C, 95% humidity and 5% CO_2_. Following that the selected concentration of the ethanolic extract or standard was added and cell viability was measure at 24 hr intervals for maximum of 72 hr using neutral red uptake assay as previously described.

### Statistical analysis

All data are expressed as the mean ± standard deviation (SD). The differences were considered to be statistically significant at P < 0.05. Statistical analyses were performed using Primer of Biostatistics program V5 for analysis of the unpaired Student's t-test and one-way analysis of variation (ANOVA).

## Results

**Figure 1:**
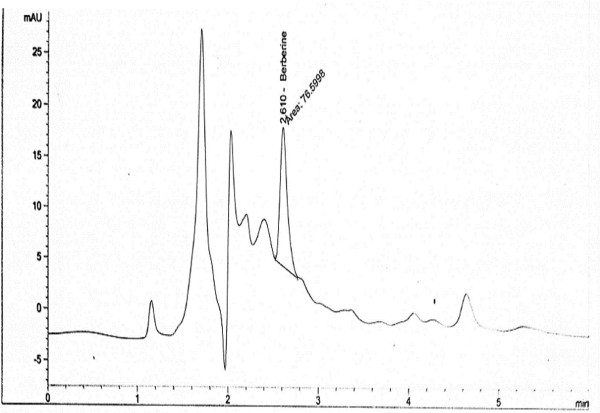
***Berberis vulgaris*** **ethanolic extract HPLC chart.**

Preliminary phytochemical screening of barberry’s roots revealed the presence of alkaloids, flavonoids, saponin, phenolic contents, terpenoids and cardiac glycosides. However, steroid and phobatannins were not detected. The percentage of alkaloids, flavonoids, saponin and total phenolic content were 4, 1.9, 0.35 g/ 100 gm plant tissue and 100 mg/ml of ethanolic extract, respectively (Table 3). Table 3 and Figure 1 showed that 1 mg of berberis ethanolic extract contains 0.6 mg berberine active compound.

**Table 3:** **Quantitative phytochemical screening of barberry roots and Berberine concentration in** ***Berberis vulgaris*** **crude extract**

**Extract**	**Berberice concentration (mg/mg extract)**
Ethanolic extract	0.62

**Figure 2:**
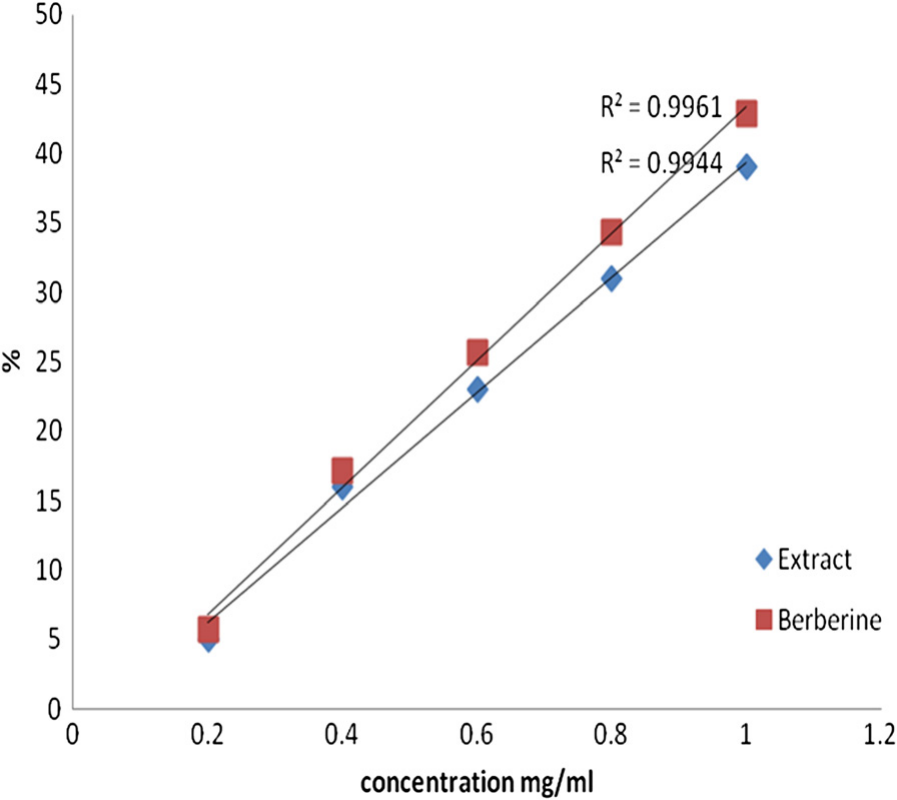
**Inhibitory effect percentage of Berberine chloride and** ***Berberis vulgaris*** **toward AChE.**

Figure 2 showed that *Berberis vulgaris* and berberine chloride different concentrations exerted the same AChE inhibitory ability in percentage (%) at *p* < 0.05, this inhibitory effect was increased as the concentration of *Berberis vulgaris* and berberine chloride increased.

**Figure 3:**
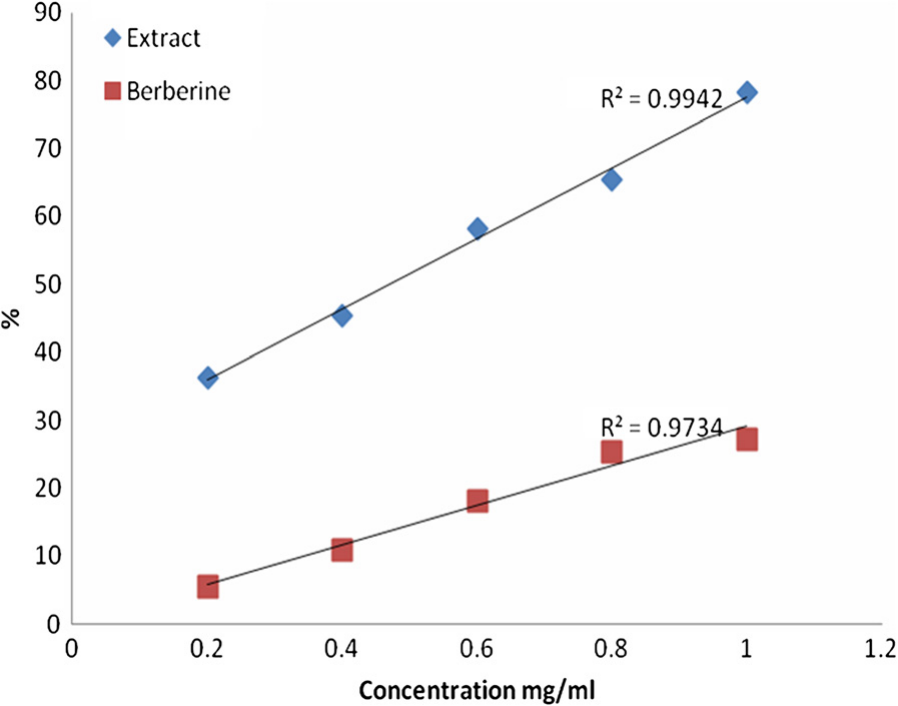
Inhibitory effect percentage of Berberine chloride and berberis vulgaris toward glucosidase.

Both *Berberis vulgaris* and berberine chloride had α-glucosidase inhibitory effect but the effect of *Berberis* crude extract was more potent than that of berberine chloride as shown in Figure 3. Furthermore, this inhibitory effect was directly proportional with that ingredient concentration at *p* < 0.05.

The antioxidants effect of Berberis vulgaris crude extract and berberine chloride were tabulated in Table 4. Our results showed that both crude extract and active ingredient had powerful antioxidants properties as they inhibited the production of TBARS, NO and the oxidation of DPPH that associated with GPx and SOD hyperactivation. These biochemical properties were exerted in a concentration dependent manner where TBARS production decreased from 9 ± 0.3 nmol/g to 4 ± 1.1 nmol/g as the concentration of *berberis* crude extract increased from 0.2 mg/ml to 1 mg/ml. The same pattern and the same inhibitory effect were shown with berberine chloride different concentrations. Both *berberis* extract and berberine chloride different concentrations ranged from 0.2-1 mg/ml lowered NO level in range from 16 to 25%, respectively, at *p* < 0.05. Furthermore, the same tested concentrations of *B. vulgaris* or berberine chloride inhibited the DPPH oxidation in range from 13 to 46% than control level at *p* < 0.05. On the other hand, the GPx and SOD activities increased in the range of 10-70% and 55-270%, respectively, when the concentration of *Berberis vulgaris* or berberine chloride increased from 0.2 mg/ml to 1 mg/ml as comparing with control levels.

**Table 4:** Effect of berberine chloride and berberis on cellular prooxidants/antioxidants status

**mg/rn**	**Tears**		**DPPH**		**NO**		**Gpx**		**SOD**	
1										
	Extract	Berberine	Extract	Berberine	Extract	Berberine	Extract	Berberine	Extract	Berberine
0	12 ± 2.5		22 ± 5.1		60 ± 12		0.3 ± 0.02		2.5 ± 0.2	
0.2	9 ± 0.3	8 ± 0.7	19 ± 2.1	19 ± 0.1	50 ± 1.1	55 ± 1	0.33 ± 0.0 1	0.39 ± 0.0 5	3.9 ± 0.1	4 ± 0.3
0.4	7 ± 0.7	6 ± 0.1	17.1.1	16 ± 0.1	50 ± 2.1	53 ± 2.1	0.38 ± 0.0 3	0.42 ± 0.0 3	*5 ± 0.3*	5.1 ± 0.5
0.6	4.5 ± 0.4	5.3 ± 0.6	15 ± 0.9	14 ± 0.3	48 ± 1.9	49 ± 0.2	0.43 ± 0.0 2	0.48 ± 0.0 2	6.6 ± 0.2	6 ± 0.6
0.8	4.4 ± 0.8	4.5 ± 0.3	12.3 ± 0.5	12 ± 1.1	46 ± 2	46 ± 0.2	0.49 ± 0.0 2	0.52 ± 0,0 1	8.9 ± 0.4	9 ± 0.4
1	4 ± 1.1	4 ± 0.6	12 ± 2.3	11 ± 1.6	45 ± 9	43 ± 8.1	0.5 ± 0.05	0.55 ± 0.0 5	9.2 ± 0.9	10 ± 0.5

**Figure 4:**
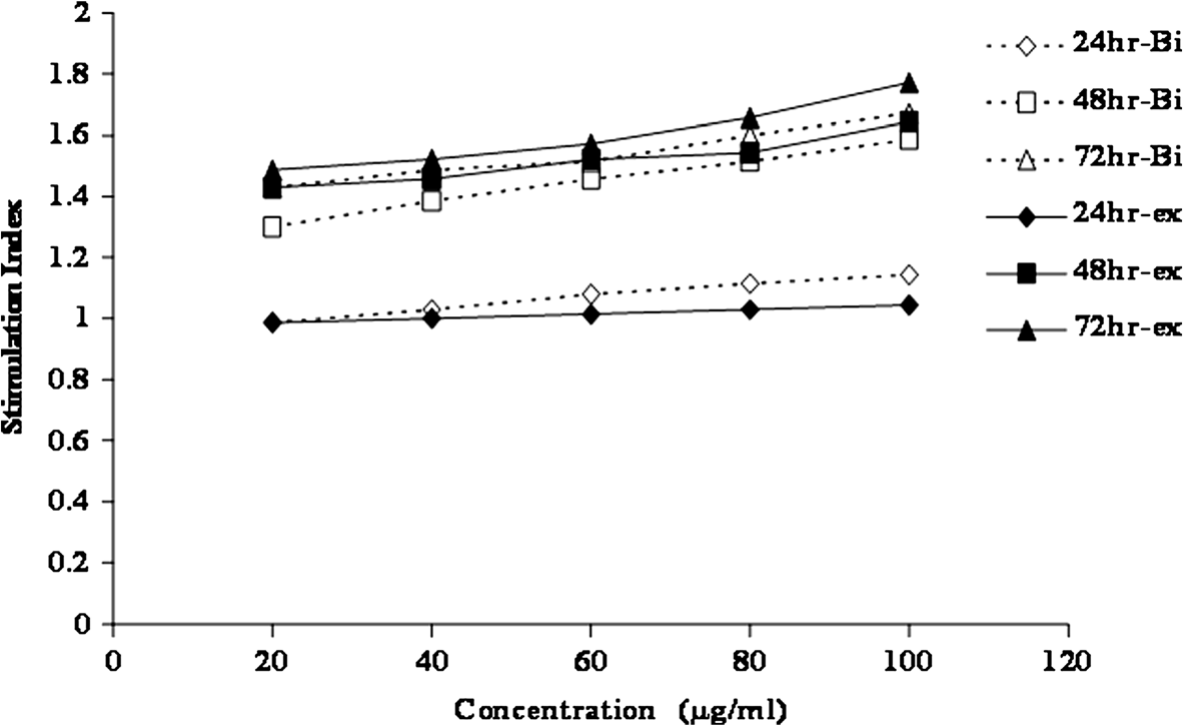
**Effect of different concentrations of Berberine chloride (Bi) and*Berberis vulgaris*extract*(ex)*on proliferation of normal peripheral blood mono nuclear cells (PBMC) at 24, 48 and 72 hrs.** Data were represented as stimulation index (SI).

**Figure 5:**
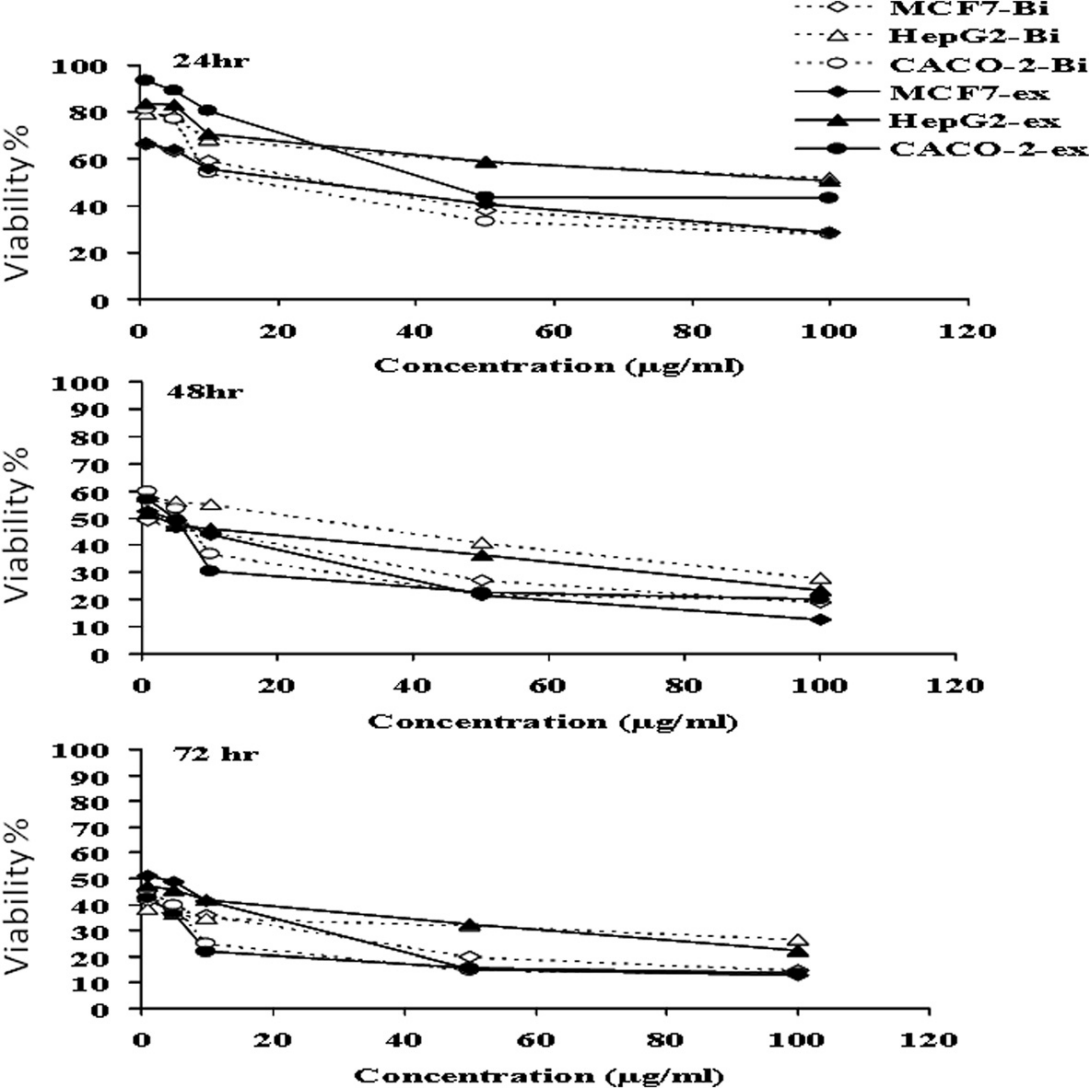
**Effect of Berberine chloride (Bi) and*Berberis vulgaris*extract*(ex)*on viability of three human cancer cell lines.** Different concentration of both Berberine chloride and berberis vulgaris were incubated with 10^5^ cells /ml of breast cancer (MCF7), Liver Cancer (HepG2) and Colon cancer (CACO-2) cell lines (ATTC, see Methods) for 24, 48 and 72 hours, cell viability were evaluated by using neutral red cell stainig.

The cytotoxicity study that was done on PBMC for both of *b. vulgaris* extract and berberine chloride, the main alkaloid constituent, showed that both of them had not any mentioned cellular toxicity while they had a significant proliferatory effect. As, different concentrations (20, 40, 60, 80 and 100 μg/ml) of both berberine chloride and barberry ethanolic extract showed to have inhibitory effect on normal blood cells (PBMC) growth rate maintenance, in contrary they slightly stimulated the proliferation of PBMC (Figure 4) especially after incubation for 72 hrs. At the same time as indicated in Figure 5, concentrations starting from 1 μg/ml up to 100 μg/ml of both berberine chloride and barberry ethanolic extract showed to have inhibitory effect on the growth of breast, liver and colon cancer cell lines (MCF7, HepG2 and CACO-2, respectively) at different incubation times starting from 24 hrs up to 72 hrs and the inhibitory effect increased with time in a dose dependant manner. It was interest to notice that with time the inhibitory dose of both berberine chloride and barberry ethanolic extract increased with time in case of normal cells (PBMC) and decreased dramatically with time in case of cancer cells (Table 5).

**Table 5:** **Inhibition concentrations of both Berberine chloride and** ***Berberis vulgaris*** **that can inhibit the growth of 50% of normal and cancer cells at different incubation time**

**11250 (mg/mi)**						
	**Berberine**			***Berheris vuigaris***		
	**24**	**48**	**72**	**24**	**48**	**72**
PBMC	0.66649	5.568	883 .994	0.90 19	6.05 1	2.546
MCF7	0.01593	0.00443	0.00195	0.01561	0.00454	0.00397
Rep 02	0.06586	0.0 1149	0.00 173	0.06805	0.00555	0.00408
CAC 0-2	0.0 1764	0.005 1	0.00 183	0.04996	0.00384	0.00 144

## Discussion

The phytochemical constituents of *B. vulgaris* act in synergism to increase barberry's bioactivity such as antioxidant, antimicrobial, anticholinergic, anti-diabetic, etc. [41]. In our previously published work, we mentioned that *B. vulgaris* ethanolic extract has a competitive AChE inhibitory ability suggesting its use to alleviate over activity of AChE in dementia patients [42]. In agreement with this finding, our data showed the same inhibitory effect toward AChE enzyme. This inhibitory effect may be returned to the presence of berberine in ethanolic crude extract where it represented 60% of its ingredients. Berberine binds AChE active site as it is acts as competitive inhibitor that leads to enzyme conformational change and increases entropy value. AChE is mainly present in the central nervous system and its principle role is to catalyze the hydrolysis of the neurotransmitter acetylcholine (ACh) to choline. This process can return an activated cholinergic neuron back to its resting state. The pathogenesis of AD is linked to a deficiency in the brain ACh [32]. Thus, AChE is an important pathogenic factor of AD and most pharmacological studies for screening agent to combat AD has been focused on AChE inhibitors to alleviate cholinergic deficit and improve neurotransmission [43].

*B. vulgaris* inhibited α-glucosidase enzyme activity provides an effective way for diabetes treatment. The inhibition of α -glucosidase activity is one of therapeutic approaches for reducing postprandial hyperglycemia. α-Glucosidase inhibitor is effective in delaying absorption of carbohydrates and suppressing postprandial hyperglycemia which contribute to the decrease in hemoglobin A1C (HbA1c). The decreasing of HbA1c could reduce the incidence of chronic vascular complication in diabetic patients [44].

Both of TBARS and DPPH assays besides SOD and GPx activities give a complete picture about the total antioxidants capacity of the *B. vulgaris* ethanolic extracts. It is important to determine the relative antioxidant capacity of the extract since free radicals and oxidants in the body commonly cause damage to aqueous-based cellular structures and organelles as well as lead to peroxidation of lipids [45]. *Berberis vulgaris* ethanolic extract was a potent inhibitor for hepatocytes' lipid peroxidation induced by Fe^2+^ and H_2_O_2_. This finding was proved previously on acidic and methanolic barberry extracts [1], where berberine and barberry crude extract showed a significant reductive ability and radicals scavenging effects, especially on hydroxyl and DPPH radicals. Also, they have the ability to increase SOD and GPx activities. It is reported that the most polar solvent dissolving several compounds of different polarities such as acids, sugars or glycosides which may be contributed to the total phenolic content of the extract and represented the highly antioxidant properties. Depending on this we speculated that acetic acid extract preparations exhibited appreciable antioxidantive activity against the generation of cellular oxidized lipid particles [1].

Nitric oxide is an essential bioregulatory molecule required for several physiological processes like neural signal transmission, immune response, control vasodilatation and control of blood pressure. However, the elevation of the NO results in several pathological conditions, including cancer [46]. The plant/plant products may have the property to counteract the effect of NO formation and in turn may be of considerable interest in preventing the ill effects of excessive NO generation *in vivo*[46]*B. vulgaris* extract reduced the generation of NO *in vitro* in a concentration dependent manner. The implications of these findings may be very important for human health, since this herb has been used in several countries from ancient times. Further, the high scavenging activity may also help to arrest the chain of reactions initiated by excess generation of NO, that are detrimental to the human health as excess NO is known to damage the immune system.

Chemotherapeutic drugs generally have low safety despite of their high efficacy. This might be due to toxicity and side effects that are usually associated with almost all types of chemotherapeutic medicine, besides the generated resistance to this group of therapeutic drugs which makes it more difficult to the patients. Between 1982 and 2002, more than half of the new anti-cancer chemical compounds were derived directly or indirectly from natural resources either microorganisms, plant or both [47,48]. Berberine as an isoquinoline alkaloid isolated from different types of plants is well recognized for its assorted pharmacological actions, for example berberine purified from Coptidis Rhizoma (Huanglian) is used in Chinese medicine for heat dissipation and detoxification [49], moreover, berberine purified from members of family *Berberidaceae* is well known for its anti-inflammatory and immunosuppressive capacity beside other pharmacological activities [50-56]. Berberine is also recognized for its anti-cancer activity [56-64]. For assessment of anticancer effect of certain compounds, cytotoxicity on primary normal cell culture must be formed to calculate the safe dose. Several types of primary cells can be used; one of them is PBMC [65]. Furthermore, we based on neutral red assay for cytotoxicity study as it is reported that neutral red and the MTT assay being the most sensitive in detecting cytotoxic events compared to the LDH leakage and the protein assay [66]. Moreover, it is well known that berberine shifted the balance between Th2 and Th1, increased the production of IL 12 and altered the cytokines profile [67]. So, it mimics PHA response and in such cases PHA did not used to make cell stimulation because it could cause cell death due to cell overstimulation [68,69].

In the present study, the anti-cancer activity of berberine chloride and total ethanolic extract from *B. vulgaris* was examined, in which this herb as member of the family *Berberidaceae* has not been identified before for its anti-cancer activity. At first, the dose for this *in vitro* testing was determined using healthy PBMC, and as indicated in Figure 3, for berberine chloride and *B. vulgaris* ethanolic extracts, both are slightly stimulate immune cells and the safety margin concentrations of both berberine chloride and *B. vulgaris* ethanolic extracts are significantly high (IC_50_ =0.66649, 5.568 and 883.994 mg/ml after incubation at 24, 48 and 72 hours). Followed by examining the effect of 1, 5, 10, 50 and 100 μg/ml of both berberine chloride and *b. vulgaris* extracts on viability of breast, hepatic, colon and cervix cancer cell lines after incubation for 24, 48, and 72 hours. The cell viability of all cancer cell line used in this study was decreased significantly and in a dose dependent manner with both berberine chloride and *berberis vulgaris* ethanolic extracts (Figure 5 and Table 4). This data are in agreement with the published data about other members of family *berberidaceae*[56,58-60,64]. The mode of action of both berberine chloride and *b. vulgaris* ethanolic extracts were not determined during the course of the present study, but in another study performed in our laboratory (data not shown) that p_53_ expression was increased due to treatment with both berberine chloride and *b. vulgaris* ethanolic extracts. This might explain that *b. vulgaris* ethanolic extract can induce cancer cell death (apoptosis) through this mode of action. On the other hand, the antioxidant activity might play a major role in increasing efficiency of such extracts to kill cancer cells and protect normal cells.

## Conclusion

This work demonstrates the potential of the bioactive ingredients of barberry on suppressing lipid peroxidation, suggesting a promising use in the treatment of hepatic oxidative stress, Alzheimer and idiopathic male factor infertility. Besides*, berberis vulgaris* ethanolic extract can induce cancer cell death that could return to its powerful antioxidant activity. Although the significant curative potential of *b. vulgaris* against many diseases, it didn’t deposited in a publicly available herbarium yet. Thus, for further experimental investigations, protocol for ingredients’ extraction mentioned in methods section is recommended to be followed. Furthermore, the plant has been registered in several herbarium and we use this as reference, also we purchased the root from USA market so how come we register it in our herbarium as it is not grown in Egypt but we will register the extract after we finish complete toxicological study [31,70,71].

## Competing interests

The authors declare that they have no competing interests.

## Authors’ contributions

All authors designed the study, collected the data, performed the techniques employed in the study. GAD, AEA and EAM made the interpretation of statistical analyses and wrote the paper with input from all the authors who each approved the final version. All authors read and approved the final manuscript.

## Pre-publication history

The pre-publication history for this paper can be accessed here:

http://www.biomedcentral.com/1472-6882/13/218/prepub
